# Bone alkaline phosphatase as a surrogate marker of bone metastasis in gastric cancer patients

**DOI:** 10.1186/s12885-016-2415-x

**Published:** 2016-07-04

**Authors:** Sun Min Lim, Youn Nam Kim, Ki Hyun Park, Beodeul Kang, Hong Jae Chon, Chan Kim, Joo Hoon Kim, Sun Young Rha

**Affiliations:** Division of Medical Oncology, Department of Internal Medicine, Yonsei Cancer Center, Yonsei University College of Medicine, 50 Yonsei-ro, Seodaemun-gu, Seoul, 120-752 Republic of Korea; Division of Medical Oncology, Department of Internal Medicine, CHA Bundang Medical Center, CHA University, Seongnam, South Korea; Division of Clinical Data Management Research, Clinical Trials Center, Severance Hospital, Yonsei University Health System, Seoul, South Korea; Department of Internal Medicine, Hongik Hospital, Seoul, South Korea; Division of Hematology and Medical Oncology, Department of Internal Medicine, Seoul National University Bundang Hospital, Seongnam, South Korea; Song-dang Yonsei Cancer Research Institute, Yonsei University College of Medicine, Seoul, South Korea; Brain Korea 21 Plus Project for Medical Sciences, Seoul, South Korea

**Keywords:** Gastric cancer, Bone turnover markers, Bone metastasis

## Abstract

**Background:**

Bone metastasis is relatively uncommon in gastric cancer patients, but its incidence has been rising. Early detection of bone metastasis is important in preventing complications related to bone metastasis such as pain, fracture and the compromise of chemotherapy. In this pilot study, we investigated the feasibility of bone turnover markers as surrogate markers of bone metastasis in gastric cancer patients.

**Methods:**

Fifty-eight patients with gastric cancer were included in this study. Serum levels of bone alkaline phosphatase (ALP), parathyroid hormone (PTH), 25(OH) D, osteocalcin (OC) and C terminal telopeptide were measured and compared between patients with bone metastasis and those without. Student’s *t*-test and Mann-Whitney *U* test were used in comparing two groups, and Spearman’s rank order correlation coefficient was calculated to quantify the strength of the associations.

**Results:**

Fifty eight age- and sex-matched patients were evaluated for bone turnover markers, among whom 29 patients had bone metastasis and 29 patients with no bone metastasis. The median age was 62 and there were 20 (68.9 %) males and 9 (31.1 %) females in each group. Bone ALP was significantly higher in the patient group (57.32 ± 46.83 vs. 34.57 ± 21.57, *P* = 0.037) than control group. Bone ALP was positively associated with ALP, osteocalcin, CA19-9, CA 72–4 and negatively associated with 25(OH) D. According to ROC-curve analysis, at the threshold value of 29.60 μg/L, the sensitivity of bone ALP was 76.7 % and the specificity was 59.4 %.

**Conclusion:**

Bone ALP may be a surrogate marker of bone metastasis in gastric cancer patients. More prospective studies are warranted to determine the optimal bone turnover markers in the evaluation of bone metastasis.

**Electronic supplementary material:**

The online version of this article (doi:10.1186/s12885-016-2415-x) contains supplementary material, which is available to authorized users.

## Background

Bone metastasis occurs frequently in patients with advanced breast, prostate, lung, and kidney cancers, but is known to be less frequent in cancers arising from gastrointestinal tract [1]. Bone metastasis in gastric cancer is thus relatively uncommon, and there are only a few studies on the incidence, clinical presentation and prognosis of gastric cancer patients with bone metastasis. To date, the incidence of bone metastasis varies from 1 to 45 % from the few previous studies [2–4]. However, due to wide use of imaging diagnostics and longer survival of gastric cancer patients, the incidence of bone metastasis is increasing. The prognosis of patients with bone metastasis is very dismal [2, 5], and thus, there is a need to detect development of bone metastasis at an early stage. Since commonly used tumor markers in gastric cancers (CEA, CA 19–9, CA 72–4) cannot accurately predict the tumor burden in bone, other predictive serum markers for bone metastasis are required.

Bone metastasis results in disruption of normal homeostasis of bone, which is a dynamic process involving osteoclast-mediated osteolysis and osteoblast-mediated osteogenesis. This may result in reduced bone integrity and skeletal complications such as fracture and severe bone pain may occur [6]. Paraneoplastic syndromes such as hypercalcemia associated with bone metastasis may be life-threatening. Therefore, early detection of bone metastasis in advanced cancer patients is important for treatment and prognosis. The assessment of bone metastasis currently relies on imaging, and ^99m^Tc-based bone scintigraphy is routinely used for the detection of bone metastasis [7]. Although bone scintigraphy is highly sensitive, its specificity is low [8], and it is not cost-effective to repeat bone scintigraphy in order to detect bone metastases early in patients without bone metastasis. In addition, bone scintigraphy is not largely recommended as routine evaluation for evidence of bone metastasis at the time of diagnosis or during treatment.

The use of bone turnover markers has been investigated for diagnostic and prognostic purposes in advanced cancer patients. Bone formation markers are direct or indirect products of osteoblast activity, whereas bone resorption markers are derived from the degradation of skeletal collagen. Bone markers have been extensively studied in prostate cancers, and several studies have assessed the diagnostic efficacy of bone formation and resorption markers for detecting bone metastases [9–12]. Bone alkaline phosphatase (ALP) is an indicator of osteoblast metabolism, and it is relatively a specific marker for osteogenesis. Osteocalcin is the non-collagen protein of bone matrix, and is released during matrix synthesis and circulates in blood. C-terminal telopeptide (CTX) is a carboxy terminal peptide of mature type I collagen, and is released during bone resorption. Parathyroid hormone (PTH) may stimulate bone resorption by binding to osteoblasts to increase expression of RANKL and the binding of RANKL to RANK stimulates osteoclast, which ultimately enhances bone resorption [13]. 25(OH)D stimulates bone resorption by aiding differentiation of osteoclast progenitors to osteoclasts [14].

In this pilot study, we compared the five bone turnover markers in gastric cancer patients with or without bone metastasis. We aimed to develop surrogate measurements that might provide a useful complement to established imaging techniques for identifying patients with skeletal involvement.

## Methods

### Patient selection

This is a cross-sectional study for examining the role of bone markers in advanced gastric cancer patients from March, 2013 to March 2014 at Yonsei Cancer Center. Patient eligibility criteria were as following: 1) age ≥ 20; 2) advanced or metastatic gastric cancer patients; 3) known status of bone metastasis; 4) patients who provided informed consent. Patients were divided into two groups, one group with bone metastasis (patient group) and the other group without bone metastasis (control group) for comparison.

### Measurement of bone markers

Serum PTH was measured using an immunochemiluminescence assay for intact PTH. Serum 25(OH)D was measured by radioimmunoassay, and osteocalcin was measured by an electrochemiluminescence immunoassay method (Roche Diagnostics, Mannheim, Germany). CTx was measured by one-step ELISA and bone ALP was measured by electrochemiluminescent ELISA immunoassay (UnicelDxl 800, Beckman Coulter, USA).

### Diagnosis of bone metastasis

To identify bone metastasis, radiographic imaging modalities including radionuclide bone scintigraphy, plain radiography, CT and magnetic resonance imaging were used in all patients. Bone scitigraphy was performed 4 h after intravenous injection with ^99m^Tc-labeled methyldiphosphonate, and a gamma camera was used for recording. Simple radiographs, CT or magnetic resonance imaging were secondarily performed to confirm abnormal findings on bone scintigraphy and clinical symptoms including bone pain and paralysis. In patients with an ^2^[18^F^] Fluoro-2-deoxy-D-glucose (FDG) -PET scan showing abnormal uptake, bone scan and radiographs were performed to confirm the presence of metastatic lesions in needed.

### Statistical analysis

Patients were matched according to age and sex using propensity score matching. Patients were grouped as described above and the data was analyzed using SPSS v 20.0 software. The results were examined by comparing the value of bone markers in the groups with or without bone metastasis. For all parameters, mean ± standard deviation values were used. Paired-*t* test was performed to compare the markers between two groups, and Pearson’s correlation was calculated to quantify the strength of the associations. Sensitivity, specificity, positive predictive and negative predictive values aimed at detecting the diagnostic value of bone markers were calculated using ROC analysis. In all analysis, *P* < 0.05 was considered statistically significant.

## Results

### Baseline characteristics of patients

A total of fifty eight age- and sex-matched patients were evaluated and baseline characteristics of patient and control groups are shown in Table 1. There were 29 patients in each group and the median age was 62. There were 20 (68.9 %) males and 9 (31.1 %) females in each group. When the baseline characteristics were compared, only ECOG performance status was different between the two groups. The control group had more PS 0 patients than the patient group (*P* < 0.001). The most common site of bone metastases was pelvis (62.5 %), then vertebra (62.1 %) and rib (62.1 %), arm-shoulder (37.9 %), femur (37.9 %), cranial (3.4 %), in the order of frequency (Additional file 1: Table S1).

**Table 1:** Baseline characteristics of all patients (*N* = 58)

Characteristics	Patient (*n* = 29)	Control (*n* = 29)	*P*
Age, years (median)	62	62	1.00^a^
Sex			
Male	20	20	1.00^a^
Female	9	9	
Initial staging			0.05
I	0	1	
II	1	4	
III	5	11	
IV	23	13	
ECOG performance status			**<0.001**
0	11	24	
1	18	5	
Surgery history			0.08
Yes	13	19	
No	16	9	

^a^Age and sex-matched by propensity score matching

Significant *P*-values in bold letters

### Comparison of patient and control groups

**Fig. 1:**
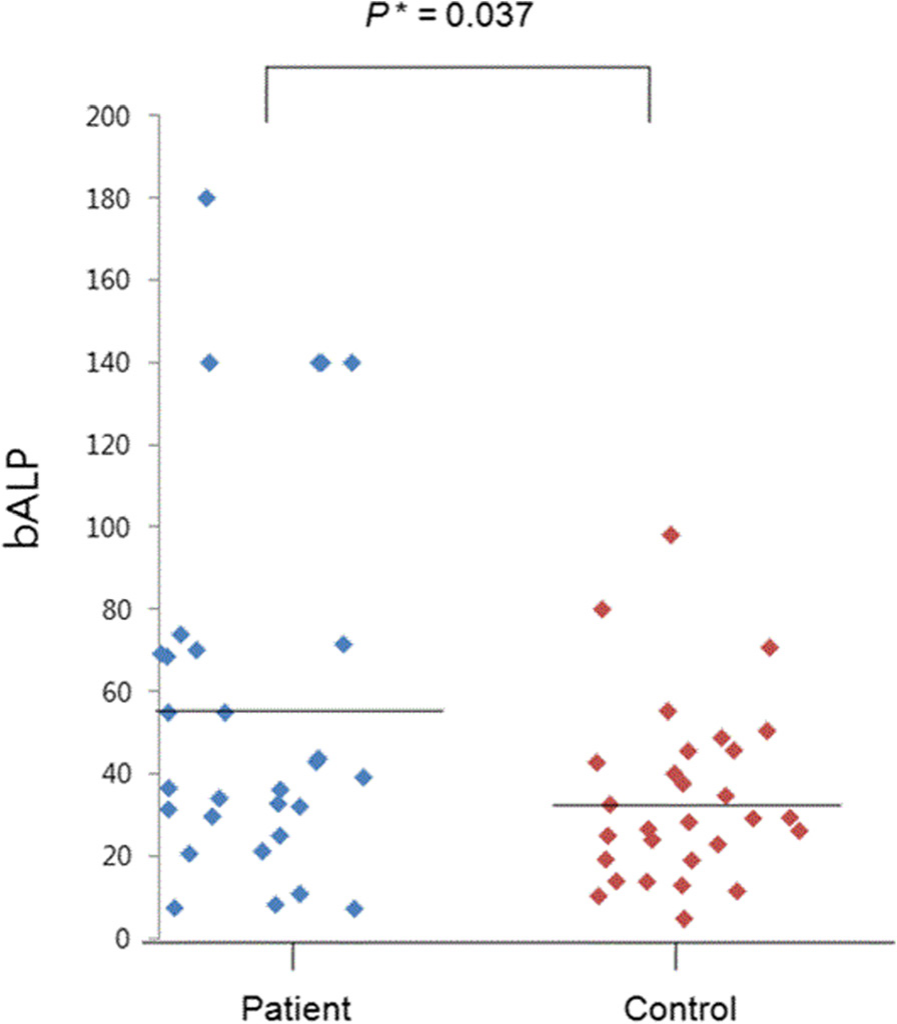
Comparison of bone ALP levels in patient and control groups. Bone ALP was significantly higher in the patient group (57.32 ± 46.83 vs. 34.57 ± 21.57, *P* = 0.037) than control group

Bone turnover markers were compared between patient and control groups (Table 2). Among the five markers, bone ALP was significantly higher in the patient group (57.32 ± 46.83 vs. 34.57 ± 21.57, *P* = 0.037) than control group. Serum osteocalcin and CTx were higher and 25(OH) D and PTH were lower in the patient group, but they did not reach a statistical significance. The comparison of bone ALP between two groups is shown in Fig. 1 as a scatter plot. When other serum markers such as LDH, calcium, CEA, CA19-9 and CA72-4 were compared between patients with bone metastasis and control groups, there were no significant differences between the two groups.

**Table 2:** Distribution of marker values between patient and control groups

Mean ± SD	Patient (*n* = 29)	Control (*n* = 29)	*P*
Osteocalcin (ng/mL)	25.55 ± 4.15	26.42 ± 3.33	0.881
Bone ALP (μg/L)	57.32 ± 46.83	34.57 ± 21.57	**0.037**
CTx (ng/mL)	1.19 ± 1.73	0.74 ± 0.32	0.193
25 (OH)D(ng/mL)	12.72 ± 7.44	12.06 ± 5.54	0.717
PTH (pg/mL)	42.97 ± 14.70	54.44 ± 57.26	0.350

Analyzed by paired *t*-test

*SD* standard deviation, *ALP* alkaline phosphatase, *CTx* serum collagen type 1 cross-linked C-telopeptide, *PTH* parathyroid hormone

Significant *P*-values in bold letters

### Correlation between bone alkaline phosphatase and serum markers

Then, we analyzed the correlation between bone ALP and other bone turnover markers and serum markers. Bone ALP was positively associated with ALP, osteocalcin, CA19-9, CA72-4 and negatively associated with 25(OH) D. There was no significant correlation between bone ALP with CTx, PTH, LDH, calcium and CEA level (Table 3).

**Table 3:** Correlation between bone ALP and other serum markers

	ALP		Osteocalcin		CTx		25(OH) D		PTH		LDH		Calcium		CEA		CA19-9		CA72-4	
	c	*P*	c	*P*	c	*P*	c	*P*	c	*P*	c	*P*	c	*P*	c	*P*	c	*P*	c	*P*
Bone ALP	0.584	<**0.001**	0.335	**0.011**	−0.022	0.867	−0.315	**0.022**	0.253	0.065	−0.031	0.821	0.016	0.904	0.161	0.226	0.342	**0.009**	0.344	**0.011**

Analyzed by Spearman’s correlation

*c* correlation coefficient

Significant *P*-values in bold letters

We also analyzed correlation between bone ALP and SUV uptake by PET-CT. With 13 patients who underwent PET-CT at diagnosis, we could not find a significant relationship between serum levels of bone ALP and the maximum value of FDG uptake (Pearson’s correlate coefficient 0.161, *P* = 0.60).

### Diagnostic performance of bone markers

**Fig. 2:**
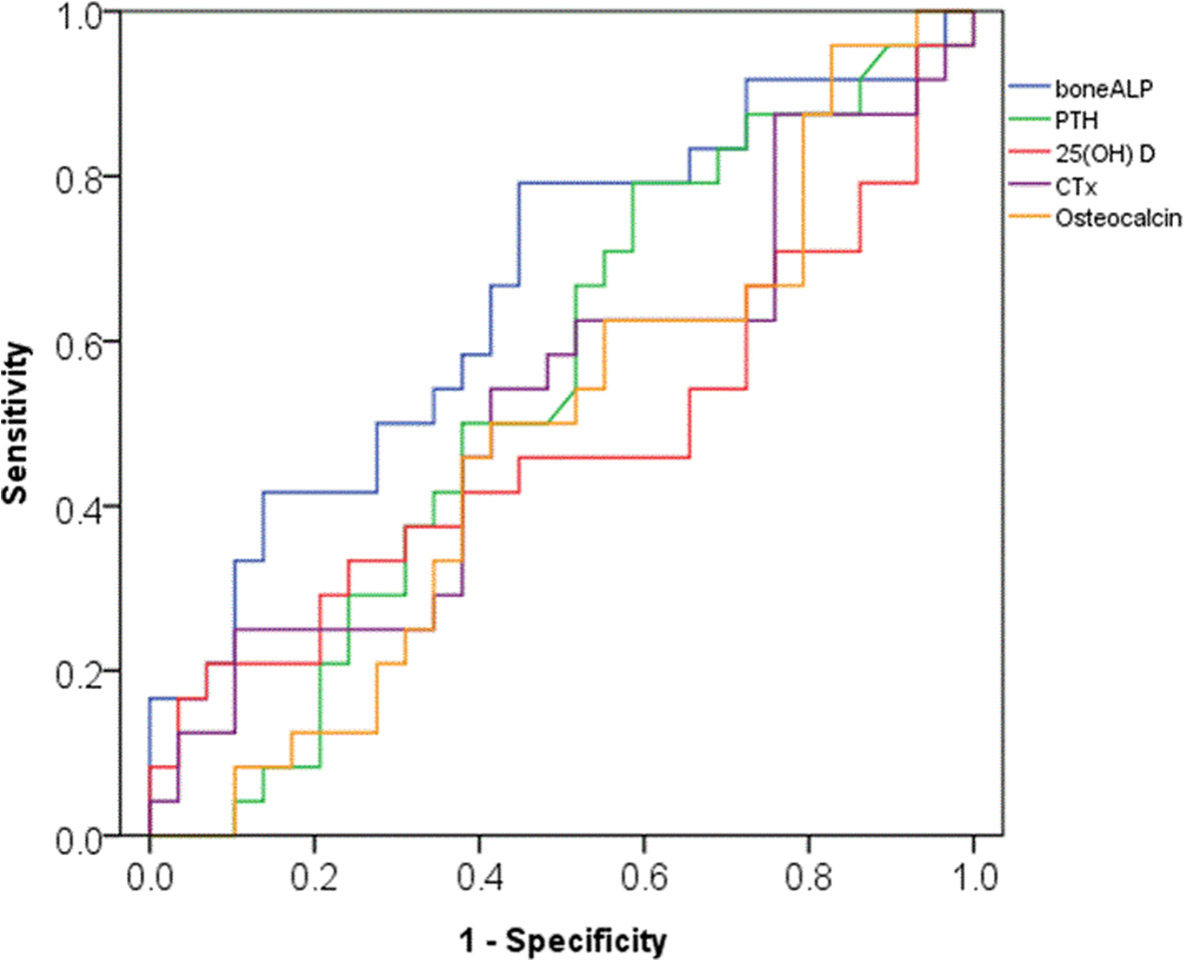
Receiver operating characteristics curves of bone metastasis according to five bone turnover markers. The best overall diagnostic performance for discriminating patients with bone metastases was provided by bone ALP assay, followed closely by PTH and CTx, and then by osteocalcin and 25(OH) D, in the order of diagnostic validity. The area under the ROC curve was 0.662, 0.542, 0.520, 0.484, and 0.474, respectively

The best overall diagnostic performance for discriminating patients with bone metastases was provided by bone ALP assay, followed closely by PTH and CTx, and then by osteocalcin and 25(OH) D, in the order of diagnostic validity. The area under the ROC curve was 0.662, 0.542, 0.520, 0.484, and 0.474, respectively (Fig. 2). According to ROC-curve analysis, the sensitivity of bone ALP was 76.7 % and the specificity was 59.4 % at the threshold value of 29.60 μg/L. When bone ALP values were dichotomized by the threshold value, there were 24 patients (82.7 %) with high bone ALP values in the patient group, whereas 12 patients (41.3 %) had high values in the control group. We also examined to find the best combination of predictive markers by using logistic regression model, but addition of other bone turnover markers did not produce an addictive diagnostic value for bone metastasis.

### Patient cases

**Fig. 3:**
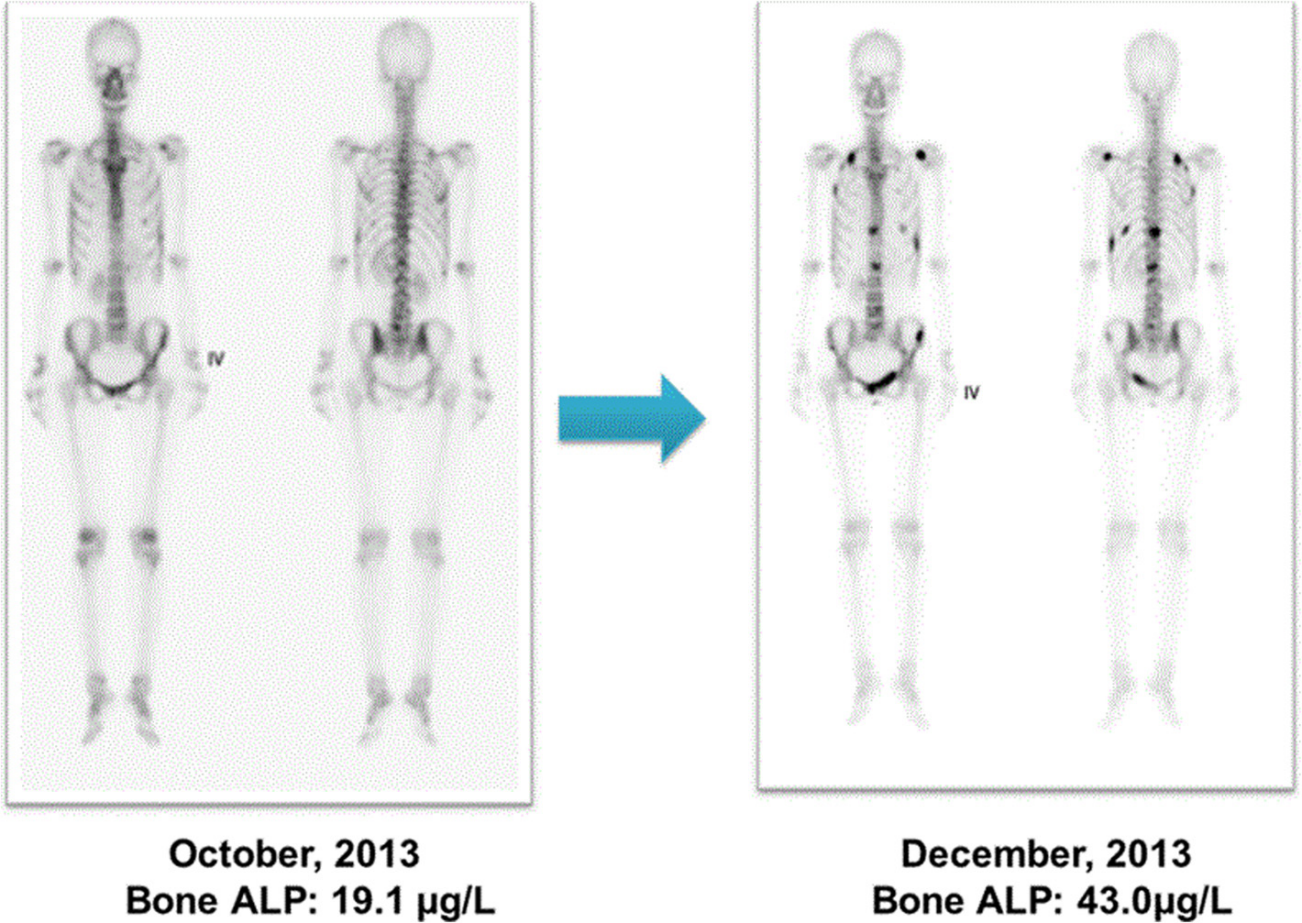
**a** A 47-year-old female patient with no known bone metastasis at baseline subsequently developed multiple bone metastases at spine and sacrum, rib cage, left scapula, and left ileum. Serum bone ALP levels increased from 19.1 μg/L to 43.0 μg/L. **b** A 64-year-old male patient initially diagnosed with multiple bone metastases showed favorable response to chemotherapy. The bone metastatic lesions showed a decrease in intensity on bone scintigraphy and bone ALP level was decreased after 3 months of chemotherapy

Here, we introduce two patient cases that suggest the feasibility of bone ALP as a predictive marker of bone metastasis and treatment response. The first patient is a 47-year-old female patient who was initially grouped as the control group without known bone metastasis. Serum bone ALP was 19.1 μg/L at baseline (May 2013), and was given palliative chemotherapy with 5-fluorouracil and docetaxel combination. When she completed 6 cycles of chemotherapy, her bone ALP was increased to 26.9 μg/L. There was no documented bone metastasis on the bone scan taken in October, 2013 and the disease was maintained stable on abdomen CT without clinical deterioration. She was continued on the chemotherapy, but when she underwent follow up imaging evaluation in December, 2013, the bone scan revealed multiple bone metastases at T-, L-spine and sacrum, bilateral rib cage, left scapula, and both left ileum. Her bone ALP increased further to 43.0 μg/L. This case shows the potential of bone ALP as an early predictor of bone metastasis in patients without documented bone metastasis (Fig. 3a).

Next is a 64-year-old male patient diagnosed with multiple bone metastases at bilateral ribs, both pelvic bones and femurs. His bone ALP was initially 43.8 μg/L. He underwent palliative chemotherapy with TS-1 plus oxaliplatin, and after 3 cycles, his tumor showed favorable response to therapy and bone metastatic lesions showed a decrease in intensity on the bone scan. In addition, bone ALP was decreased to 26.7 μg/L after 3 cycles. This case suggests that bone ALP may be a monitoring marker of response to systemic chemotherapy (Fig. 3b).

## Discussion

This is a pilot study of evaluating the feasibility of bone turnover markers for detecting bone metastasis in gastric cancer patients. To our knowledge, this is the first study to evaluate the diagnostic value of bone turnover markers in gastric cancer patients. Our hypothesis was whether bone turnover markers can differentiate patients with or without bone metastases. Bone ALP was significantly higher in the group with bone metastasis compared to those without metastasis. Bone ALP was positively associated with ALP, osteocalcin, CA19-9, CA 72–4 and was negatively associated with 25(OH) D. According to ROC-curve analysis, at the threshold value of 29.60 μg/L, the sensitivity of bone ALP was 76.7 % and the specificity was 59.4 %.

Bone ALP is one of the several isoforms of ALP that are secreted by various organs including liver, intestine and placenta [15]. It is specifically present on the surface of osteoblasts, and serum level of bone ALP was shown to have a linear relationship with osteoblast and osteoblastic precursor activity [16]. Although the diagnostic value of bone ALP has not been studied in gastric cancer, bone ALP was found significant in predicting bone metastasis in patients with breast and prostate cancer [17]. A study by Bomabardieri et al. showed that bone ALP was the best marker to discriminate bone scan positive patients from scan-negative breast cancer patients [18]. Demers LM et al. showed that bone ALP showed a significant correlation with both the presence of bone metastases and the extent of skeletal involvement in metastatic cancer patients. Moreover, levels of bone ALP were significantly higher with blastic disease presentation than with the presence of either lytic or mixed disease [19].

The two cases presented here reveal the feasibility of bone ALP as a useful complement for monitoring progression of bone disease. First case suggests that serum bone ALP level may begin to increase before bone metastasis is detectable through standard imaging techniques, and continue to rise until metastasis becomes visible. Whether bone ALP can be used to determine early bone micrometastases before shown on bone scan still remains to be determined. However, there is evidence that bone ALP might represent disease extension [19], and clinicians may infer the risks of disease progression and future skeletal complications. Second case provides prognostic information regarding the response of bone disease to systemic therapy. Thus, these findings provide evidence for the potential of bone ALP to reflect not only the presence, but also the extent of bone metastasis.

This study has a few limitations. First of all, it is a cross-sectional study and it may not reflect the continuous renewal of osteoclasts and osteoblasts. Previous studies reported that osteolytic type of bone metastasis is more common than osteoblastic or mixed type (52 vs. 22.8 vs. 25.2 %) [20], and thus the marker of osteolysis remains to be identified. Secondly, the sample size of this study is small, and a larger-scaled study is required to conclude the utility of bone ALP as compared to other bone turnover markers. Lastly, the sensitivity (76.7 %) and specificity (59.4 %) of bone ALP at the threshold value of 29.60 μg/L are not very high.

## Conclusion

In conclusion, levels of bone ALP appear to be the most predictive biochemical markers for the presence of bone metastases in gastric cancer patients. There is also preliminary evidence that the level correlates with the extent of metastatic disease. However, prospective studies are needed to examine whether this marker can be validated in a larger number of patients. In addition, future investigations are warranted to see whether bone turnover markers can be used to guide clinicians in determining the effectiveness of therapy in gastric cancer patients with bone metastases.

## Abbreviations

ALP, Alkaline phosphatase; CTX, C-terminal telopeptide; OC, osteocalcin; PTH, parathyroid hormone

## Additional file

Additional file 1: Table S1. Sites of bone metastasis. (DOCX 14 kb)
