# Supplementary material for: Bone alkaline phosphatase as a surrogate marker of bone metastasis in gastric cancer patients
Source: BMC Cancer. 2016 Jul 4;16:385. doi: 10.1186/s12885-016-2415-x (PMC4932725; doi:10.1186/s12885-016-2415-x)
Supplement: Additional file 1: Table S1. — Sites of bone metastasis. (DOCX 14 kb) [file 12885_2016_2415_MOESM1_ESM.docx]

**Supplementary Table 1. Sites of bone metastasis**

| **Location** | **No. of patients** | **%** |
| --- | --- | --- |
| Pelvis | 19 | 65.5% |
| Vertebra | 18 | 62.1% |
| Costa | 18 | 62.1% |
| Femur | 11 | 37.9% |
| Arm-shoulder | 11 | 37.9% |
| Cranial | 1 | 3.4% |
